# Gonorrhea—How to Cure It Easily and Quickly

**Published:** 1879-08-20

**Authors:** H. M. Lawson

**Affiliations:** Georgia


### *GONORRHEA—HOW TO CURE IT EASILY AND QUICKLY.*

BY DR. H. M. LAWSON, OF GEORGIA.

In the treatment of gonorrhea, I conceive that we have an unhealthy inflammation to deal with; this I convert into a healthy inflammation, by the frequent use of some weak injection (there are always indications in each case which point out the appropriate injection); after using the injection two days lay aside the syringe and give internally balsam copaiba, cubebs, or, what is infinitely superior, oxydized rosin, from our Southern pine; this and gum guiaiac in good gin, if not contra-indicated, or such combination as the medical attendant may select, for ten days, and if the patient has avoided everything known to induce relapse he is well.

The most painful and hurtful (so far as the cure of the immediate trouble is concerned) are erections and chordie; for these complications the bandage properly applied is a perfect remedy; neither is possible after the bandage is duly applied.

Before adopting these views of this disease and deducing therefrom this treatment, nothing gave me more annoyance and as little satisfaction as this class of cases; since, if I can prevail on the patient to obey instructions, a speedy cure follows almost invariably. If positive results are expected, the few directions I have given must be strictly followed.

